# Association between homologous recombination deficiency and time to treatment failure to platinum-based chemotherapy for pancreatic cancer by using the C-CAT database

**DOI:** 10.1007/s00535-024-02173-0

**Published:** 2024-11-21

**Authors:** Kazunaga Ishigaki, Yurie Tokito, Naminatsu Takahara, Hiroto Nishio, Go Endo, Koshiro Fukuda, Kota Ishida, Rintaro Fukuda, Shinya Takaoka, Hiroki Oyama, Kensaku Noguchi, Tatsunori Suzuki, Tatsuya Sato, Tomotaka Saito, Tsuyoshi Hamada, Koji Miyabayashi, Yasuyoshi Sato, Yousuke Nakai, Hidenori Kage, Katsutoshi Oda, Mitsuhiro Fujishiro

**Affiliations:** 1 Department of Clinical Oncology, The University of Tokyo Hospital, Tokyo, Japan; 2 Department of Gastroenterology, Graduate School of Medicine, The University of Tokyo, 7-3-1 Hongo Bunkyo-Ku, Tokyo, 113-8655 Japan; 3 Department of Internal Medicine, Institute of Gastroenterology, Tokyo Women’s Medical University, Tokyo, Japan; 4 Department of Respiratory Medicine, The University of Tokyo Hospital, Tokyo, Japan; 5 Department of Clinical Genomics, The University of Tokyo Hospital, Tokyo, Japan

**Keywords:** Comprehensive genomic profiling, Pancreatic cancer, Homologous recombination deficiency, Chemotherapy

## Abstract

**Background:**

Since homologous recombination deficiency (HRD) is relatively uncommon in pancreatic cancer (PC), its impact on time-to-treatment failure (TTF) among patients undergoing systemic chemotherapy for unresectable and recurrent PC remains uncertain.

**Methods:**

Among patients with unresectable and recurrent PC enrolled in the Center for Cancer Genomics and Advanced Therapeutics (C-CAT) database by July 2023, a total of 1394 patients who underwent first-line chemotherapy with either gemcitabine plus nab-paclitaxel (GnP) or FOLFIRINOX (FFX) and received tissue-based CGP tests after disease progression were included in this study. HRD was defined as the presence of germline or somatic genetic mutations in homologous recombination repair (HRR)-related genes such as *ATM, BARD1, BRIP1, BRCA1/2, CHEK2, CDK12, PALB, and RAD51C/D*. We investigated the correlation between HRD and TTF among patients treated with GnP and FFX.

**Results:**

First-line chemotherapy consisted of GnP in 69% of the cases and FFX in 31%. The CGP tests used were NCC OncoPanel and FoundationOne CDx in 26% and 74%, respectively. HRR-related genetic abnormalities were identified in 107 patients (7.6%): *BRCA2* (*n* = 51), *ATM* (*n* = 34), *BRCA1* (*n* = 9), *PALB2* (*n* = 9), among others. In the GnP cohort, the median TTF was comparable between the HRD and non-HRD groups (5.3 vs 4.6 months, *P* = 0.44). Conversely, in the FFX cohort, it was significantly longer in the HRD group compared to the non-HRD group (7.3 vs. 4.7 months, *p* < 0.01).

**Conclusions:**

Our findings suggest that HRR-related genetic abnormalities might be predictive of TTF in platinum-based chemotherapy for PC.

**Supplementary Information:**

The online version contains supplementary material available at 10.1007/s00535-024-02173-0.

## Introduction

The comprehensive genomic profiling (CGP) tests have been covered by insurance in Japan since June 2019 [1, 2]. Since then, the number of patients with solid tumors undergoing CGP test has been increasing every year. In June 2018, the Japanese government established the Center for Cancer Genomics and Advanced Therapeutics (C-CAT) [3] to support hospitals engaged in cancer genome medicine. C-CAT’s primary role is to collect genomic and clinical information from all patients who have undergone CGP tests, facilitating the appropriate secondary use of the centralized information for future innovative research in Japan. Research utilizing the C-CAT database have been conducted in various cancers, ranging from investigations into genetic abnormalities in rare cancers like salivary gland carcinoma and soft tissue sarcoma, to analyses of genetic abnormality trends based on histological subtypes and the efficacy of chemotherapy in major cancers such as pancreatic cancer (PC) [4–13].

In unresectable and recurrent PC, the combination therapy with gemcitabine and nab-paclitaxel (GnP) [14] and FOLFIRINOX (FFX) [15] are established as a standard first-line chemotherapy. The GENERATE study was a randomized Phase II/III trial targeting patients with metastatic or recurrent PC in Japan [16]. This study aimed to assess the superiority of mFFX and combination therapy with S-1, oxaliplatin, and irinotecan (S-IROX) compared to the standard treatment of GnP. However, the interim analysis revealed that overall survival (OS) was significantly shorter in the mFFX and S-IROX groups compared to the GnP group. Consequently, the trial was terminated early based on the recommendation from the efficacy and safety evaluation committee, and GnP remains the standard first-line treatment for metastatic or recurrent PC in Japan.

Meanwhile, a regimen containing platinum agents followed by maintenance therapy with a poly ADP-ribose polymerase (PARP) inhibitor can be a standard of care in patients with germline *BRCA1/2* mutations, observed in approximately 4–7% of PC patients [17]. The mutations in homologous recombination repair (HRR)-related genes such as *BRCA1/2* and *PALB2* lead to defective homologous recombination repair function (Homologous recombination deficiency; HRD), which renders platinum agents and PARP inhibitors effective for treatmnet [18–20]. In addition to *BRCA1/2* and *PALB2*, there are several other HRR-related genes that may play important roles in DNA repair process [21]. However, the relationship between mutations in these genes and the efficacy of platinum agents in unresectable and recurrent PC remains unclear. Therefore, we aimed to analyze the clinical and genomic characteristics of patients with unresectable and recurrent PC, and to analyze the association between HRD and response to platinum-based regimens using the nationwide, large-scale C-CAT database in Japan.

## Methods

### Study population

We conducted this retrospective observational study using data from the C-CAT database. The database contains clinical and genomic data, clinical information such as age, sex, cancer type, pathological diagnosis, and metastatic organs. Moreover, it contains details about chemotherapy regimens including the start and end dates of treatment for each regimen, antitumor response and occurrence of severe adverse events (AEs) during chemotherapy before undergoing CGP test. Additionally, C-CAT database includes information on potential clinical trials based on findings from CGP tests. Genomic information includes the type of genes detected to be abnormal, the mutation type, gene mutation frequency and clinical significance, microsatellite instability (MSI) and tumor mutational burden (TMB) status.

This study included patients with unresectable and recurrent PC who underwent GnP or FFX (including both the original and modified regimens) as first-line treatment, and whose data were entered into the C-CAT database from June 2019 to March 2023. The cases with insufficient data on the start and end dates of first-line treatment were excluded from this analysis. In this study, only two tissue panels, Oncoguide^™^ NCC oncopanel (NOP) and FoundationOne® CDx (F1CDx), were included. In liquid-based panels, circulating tumor DNA may not be successfully detected depending on the tumor status. Additionally, the detection of clonal hematopoiesis of indeterminate potential (CHIP) is common, making it difficult to distinguish from tumor-derived genetic abnormalities. Due to the risk of mistaking CHIP for tumor-derived genetic abnormalities in this study, liquid-based panels were excluded. This study was approved by our institutional ethics committee (# 2021341G) and the Information Utilization Review Board of C-CAT (# CDU2022-026N).

### Outcomes

To assess treatment effectiveness, the overall response rate (ORR), disease control rate (DCR), time to treatment failure (TTF), and OS were analyzed. ORR was defined as the percentage of complete response (CR) and partial response (PR), while DCR was defined as the percentage of CR, PR, or stable disease (SD) among all enrolled patients. TTF was defined as the time from treatment initiation to the date of treatment discontinuation or death. The C-CAT database includes cases where the reasons for treatment discontinuation are unclear, whether due to AEs or tumor progression. However, information on the start and end dates of the treatment are accurately recorded in all cases, we adopted TTF instead of progression free survival as a reliable endpoint. Genomic information was collected on representative genetic mutations associated with PC and HRD. HRD was defined as having germline or somatic genetic abnormalities in *ATM, BARD1, BRIP1, BRCA1/2, CHEK2, CDK12, PALB, and RAD51C/D*. Although there is a difference in the number of genes analyzed in F1CDx and NOP (324 genes and 124 genes, respectively), both CGP tests include the ten HRR-related genes targeted in this study. The evidence level of C-CAT findings, graded from A to F, was used to define the pathological genetic abnormalities. Breast, ovarian, pancreatic, and prostate cancers were defined as HRD-associated cancers and the correlation between a family history of HRD-related cancers and HRD was investigated. Due to the nature of the database, which is based on data manually entered by each attending physician, some patients were found to have missing data. Therefore, the percentages for each variable were based on the number of patients with available data. In the C-CAT database, unknown information is categorized as “unknown,” and the frequency of missing values for the variables used in this analysis was relatively low, was as low as 0.1% to 0.3%.

### Statistical analysis

Continuous variables are presented as medians and ranges, and categorical variables as numbers and percentages. Statistical comparisons were performed using Mann**–**Whitney U test for continuous variables and the chi-square test or Fisher’s exact test for categorical variables. TTF and OS were estimated with 95% confidence intervals (CI) using the Kaplan–Meier method and compared using the log-rank test. We investigated the association between HRD and TTF in GnP and FFX. As a subgroup analysis, *BRCA1/2* and *PALB2*, which have a high germline conversion rate (GCR) [22], were analyzed separately from other HRR-related genetic abnormalities. We conducted a three-group comparison among those with HRD harboring mutations in *BRCA1/2* or *PALB2 (BRCA/PALB* group*)*, those with HRD harboring mutations in genes other than *BRCA1/2* or *PALB2* (Other HRR-related gene mutations group: Other HRRm group*)*, and the non-HRD group. All statistical tests were two-tailed and the significance level was set at P value of 0.05. The R software (version 2.14.0; R Development Core Team: http://www.r-project.org) was used for all statistical analyses.

## Results

### Patient characteristics

Patient characteristics are shown in Tables 1 and 2. During the study period, a total of 1394 patients with unresectable and recurrent PC underwent CGP tests. Of these, 803 were male (58%), with a median (range) age of 64 (20–86) years. Histological subtypes included adenocarcinoma in 1273 patients, adenosquamous carcinoma in 33 patients, acinar cell carcinoma in 25 patients, anaplastic carcinoma in 7 patients, and other subtypes in 56 patients, respectively. Patients with distant metastases were observed in 1257 patients (90%). The family history of cancer was present in 1070 patients (77%), and 118 patients (8%) had a history of multiple cancers. The CGP tests used were F1CDx in 1030 patients (74%) and NOP in 364 patients (26%). Tissue samples submitted consisted of surgical specimens in 481 patients, biopsy specimens in 907 patients, and 6 patients were of unknown origin. The primary treatment consisted of GnP in 957 patients (69%) and FFX in 437 patients (31%). Comparison between the HRD group and non-HRD group showed a higher prevalence of acinar cell carcinoma in the HRD group (*P* = 0.049). Although the HRD group tended to be younger and have more patients with a family history of cancer, the differences were not statistically significant. When comparing regimens, the FFX cohort was significantly younger and had a higher proportion of males. In the GnP cohort, there was a significant increase in the submission of surgical specimens, resulting in a significantly higher rate of GnP therapy for postoperative recurrence. In the GnP cohort, 2nd line chemotherapy introduction rate was similarly high in the HRD and non-HRD groups (93% and 90%, *p* = 0.54). Furthermore, the frequency of using platinum regimens in second-line and later treatment was comparable between the HRD group (59%) and the non-HRD group (52%) (Supplementary Table 1).

**Table 1:** Patient characteristics according to HRD status

		Total	HRD	Non-HRD	
		*N* = 1394	*N* = 107	*N* = 1287	*P* value
Sex	Male, *n* (%)	803 (58)	57 (53)	746 (58)	0.36
Age	Median (range), years	64 (20–86)	62 (20–81)	64 (25–86)	0.09
ECOG PS	0/1/2 < = , *n*	845/507/42	64/42/1	781/465/41	0.63
Smoking	Yes, n (%)	706 (51)	49 (46)	657 (51)	0.36
Histology	Adenocarcinoma, *n* (%)	1273 (91)	95 (89)	1178 (92)	0.049
	Adenosquamous carcinoma, *n* (%)	33 (2)	1 (1)	32 (3)	
	Acinar cell carcinoma, *n* (%)	25 (2)	7 (7)	18 (1)	
	Anaplastic carcinoma, n (%)	7 (1)	0 (0)	7 (1)	
	Others, *n* (%)	56 (4)	4 (4)	52 (4)	
Family history of cancer	Yes, *n* (%)	1070 (77)	88 (82)	982 (76)	0.07
Double cancer (different organs)	Yes, *n* (%)	118 (8)	13 (12)	105 (8)	0.28
Sample method	Surgery/Biopsy/Unknown, *n*	481 (35)/907 (65)/6 (0.4)	36 (34)/69 (64)/2 (2)	445 (35)/838 (65)/4 (0.3)	0.12
Sample area	Primary/metastatic/Unknown site, *n*	890 (64)/503 (36)/1 (0.1)	59 (55)/48 (45)/0 (0)	831 (65)/455 (35)/1 (0.1)	0.13
CGP test	F1CDx/NOP, *n*	1030 (74)/364 (26)	86 (80)/21 (20)	944 (73)/343 (27)	0.14
Metastatic	Yes, *n* (%)	1257 (90)	100 (94)	1157 (90)	0.70
First-line chemotherapy	FFX, *n* (%)	437 (31)	34 (32)	403 (31)	0.91
	GnP, *n* (%)	957 (69)	73 (68)	884 (69)	

*ECOG PS* Eastern Cooperative Oncology Group performance status, *CGP* comprehensive genomic profiling, *F1CDx* FoundationOne^®^ CDx, *NOP* Oncoguide^™^ NCC oncopanel, *FFX* FOLFIRINOX, *GnP* gemcitabine and nab-paclitaxel, *HRD* homologous recombination deficiency

**Table 2:** Patient characteristics according to first-line regimen

		Total	FFX	GnP	
		*N* = 1394	*N* = 437	*N* = 957	*P* value
Sex	Male, *n* (%)	803 (58)	276 (63)	527 (55)	< 0.01
Age	Median (range), years	64 (20–86)	61 (26–83)	66 (20–86)	< 0.01
ECOG PS	0/1/2 < = , *n*	845/507/42	265/156/16	580/351/26	0.73
Smoking	Yes, *n* (%)	706 (51)	229 (53)	477 (50)	0.24
Histology	Adenocarcinoma, *n* (%)	1273 (91)	397 (91)	876 (92)	0.74
	Adenosquamous carcinoma, *n* (%)	33 (2)	12 (3)	21 (2)	
	Acinar cell carcinoma, *n* (%)	25 (2)	9 (2)	16 (2)	
	Anaplastic carcinoma, *n* (%)	7 (1)	2 (1)	5 (1)	
	Others, *n* (%)	56 (4)	17 (4)	39 (4)	
Family history of cancer	Yes, *n* (%)	1070 (77)	348 (80)	722 (76)	0.15
Double cancer (different organs)	Yes, *n* (%)	118 (8)	31 (7)	87 (9)	0.39
Sample method	Surgery/Biopsy/Unknown, n	481 (35)/907 (65)/6 (0.4)	117 (27)/317 (73)/3 (0.6)	364 (38)/590 (62)/3 (0.3)	< 0.01
Sample area	Primary/metastatic site, *n*	890 (64)/503 (36)/1 (0.1)	253 (58)/184 (42)/0 (0)	637 (67)/319 (33)/1 (0.1)	< 0.01
CGP test	F1CDx/NOP, *n*	1030 (74)/364 (26)	324 (74)/113 (26)	706 (74)/251 (26)	0.90
Metastatic	Yes, *n* (%)	1257 (90)	391 (90)	866 (91)	0.60
HRR-related gene abnormality	Yes, *n* (%)	107 (8)	34 (8)	73 (8)	0.91

*ECOG PS* Eastern Cooperative Oncology Group performance status, *CGP* comprehensive genomic profiling, *HRR* homologous recombination repair, *F1CDx* FoundationOne^®^ CDx, *NOP* Oncoguide^™^ NCC oncopanel, *GnP* gemcitabine and nab-paclitaxel, *FFX* FOLFIRINOX

### Gene abnormalities

Details of major gene abnormalities in PC are shown in Table 3. Among the 1394 patients, mutations in *KRAS, TP53, CDKN2A, SMAD4* were observed in 1266 (91%), 1026 (74%), 262 (19%), 245 (18%), respectively. When comparing the HRD group and the non-HRD group, mutations in *KRAS*, *TP53*, and *CDKN2A* were significantly more common in the non-HRD group. Six patients (0.4%) exhibited MSI-high, and 39 patients (2.8%) exhibited TMB-high. The median (range) TMB was 2.5 (0–231.8) mut/Mb. Patients with TMB-high were significantly more frequent in the HRD group (10.3% vs 2.2%, *P* < 0.01), and median TMB was also significantly higher in the HRD group (3.0 mut/Mb vs 2.5 mut/Mb, *P* < 0.01). In the HRD group, *BRCA2* was the most common mutation observed in 51 patients (47.7%), followed by *ATM* in 34 patients (31.8%), and *BRCA1/PALB2* in 9 patients each (8.4%).

**Table 3:** Major gene abnormality

	Total	HRD	Non-HRD	
	*N* = 1394	*N* = 107	*N* = 1287	*P* value
Major pathogenic mutations				
*KRAS*, *n* (%)	1266 (91)	91 (85)	1175 (91)	0.04
* TP53*, *n* (%)	1026 (74)	53 (50)	973 (76)	< 0.01
*CDKN2A*, *n* (%)	262 (19)	12 (11)	250 (19)	0.04
*SMAD4*, *n* (%)	245 (18)	13 (12)	232 (18)	0.15
MSI-high, *n* (%)	6 (0.4)	1 (1.1)	5 (0.5)	0.43
HRR-related gene abnormality (both somatic and germline)				
*BRCA/PALB* group, *n* (%)		69 (64.4)		
*BRCA1*, *n* (%)		9 (8.4)		
*BRCA2*, *n* (%)		51 (47.7)		
*PALB2*, *n* (%)		9 (8.4)		
Other HRRm group, *n* (%)		46 (43.0)		
*ATM*, *n* (%)		34 (31.8)		
*BARD1*, *n* (%)		2 (1.9)		
*BRIP1*, *n* (%)		3 (2.8)		
*CDK12*, *n* (%)		1 (0.9)		
*CHEK2*, *n* (%)		3 (2.8)		
*RAD51C*, *n* (%)		0 (0.0)		
*RAD51D*, *n* (%)		3 (2.8)		
Cases with multiple HRR-related gene abnormalities, *n* (%)		7 (6.5)		
Number of HRR-related gene abnormalities per case, 2/3, *n*		6/1		
TMB, median (range), /Mb	2.5 (0–231.8)	3.0 (0–231.8)	2.5 (0–230.2)	< 0.01
TMB-high, *n* (%)	39 (2.8)	11 (10.3)	28 (2.2)	< 0.01

*HRRm* homologous recombination repair-related gene mutations, *MSI* microsatellite instability, *TMB* tumor mutation burden, *HRD* homologous recombination deficiency

### Family history of HRD-related cancers

Patients with a family history of HRD-related cancers are shown in Supplementary Table 2. A family history of HRD-related cancers, a family history of HRD-related cancers within third-degree relatives, and a family history of HRD-related cancers in individuals under 50 years old were significantly correlated with HRD.

### Association of HRD with outcomes of chemotherapy

**Fig. 1:**
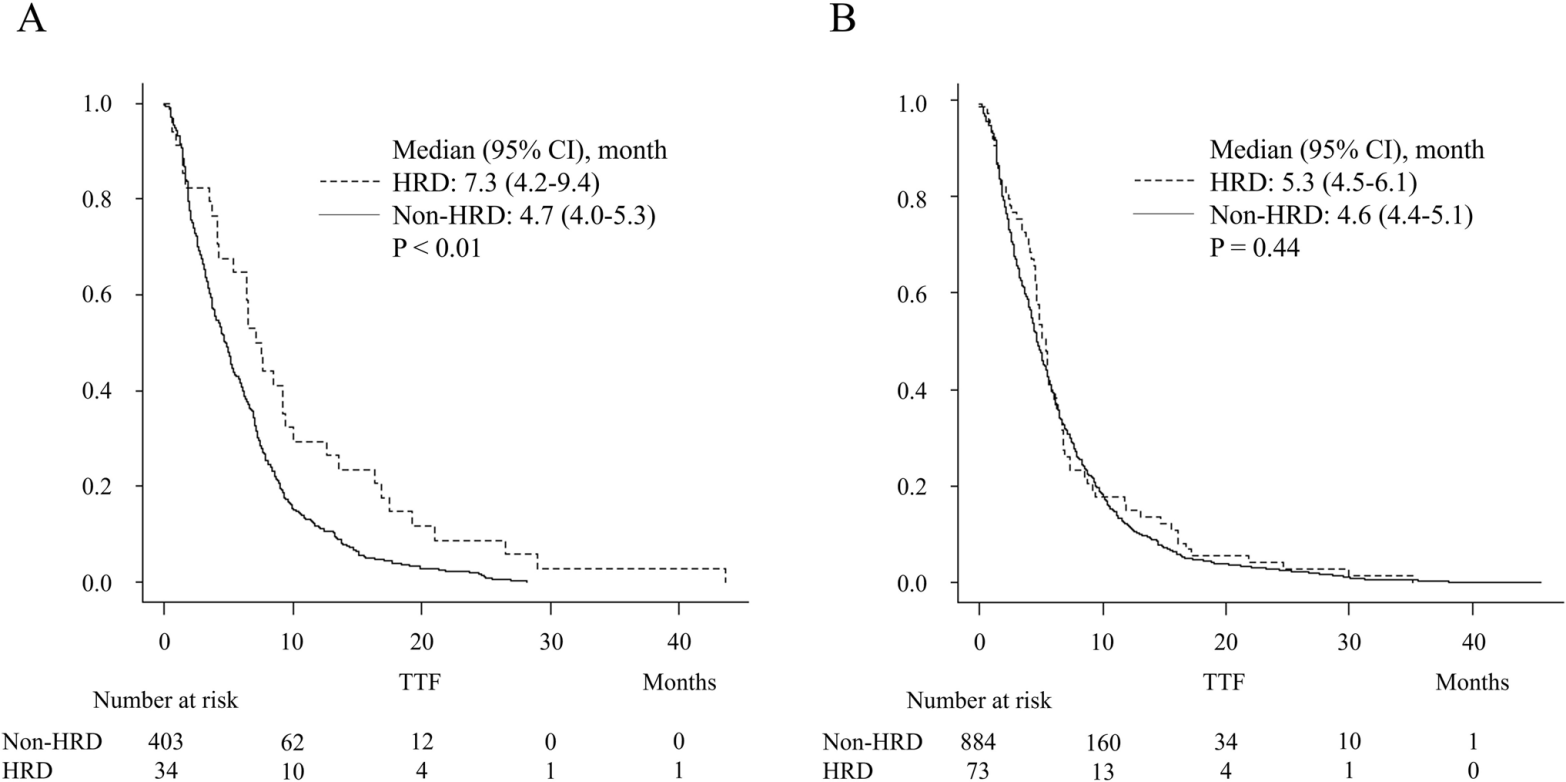
Comparison of TTF in FFX and GnP Cohort (HRD vs non-HRD). **A** In the FFX cohort, the median TTF was significantly better in the HRD group, with a median of 7.3 (95% CI: 4.2–9.4) months compared with 4.7 (95% CI: 4–5.3) months in the non-HRD group. **B** In the GnP cohort, the median TTF was comparable between the HRD group (5.3 months, 95% CI: 4.5–6.1 months) and the non-HRD group (4.6 months, 95% CI: 4.4–5.1 months). *TTF* time-to-treatment failure, *FFX* FOLFIRINOX, *GnP*;gemcitabine and nab-paclitaxel, *HRD* homologous recombination deficiency, *CI* confidence interval

The efficacy of the platinum-based regimen is shown in Table 4. In the FFX cohort, ORR and DCR were 33% and 66%, respectively. ORR in the HRD group was 56%, significantly higher than the 32% observed in the non-HRD group (*P* = 0.01). While DCR was favorable in the HRD group, there was no significant difference between the two groups (*P* = 0.26). The median TTF was significantly longer in the HRD group, with a median of 7.3 (95% CI: 4.2–9.4) months compared with 4.7 (95% CI: 4.0–5.3) months in the non-HRD group (*P* < 0.01, Fig. 1a). In the GnP cohort, ORR and DCR were 33% and 74%, respectively. ORR was 32% in the HRD group and 33% in the non-HRD group, showing similar rates between both groups (*P* = 0.90). Similarly, the DCR did not significantly differ between the two groups (*P* = 0.41). The median TTF was comparable between the HRD group (5.3 months, 95% CI: 4.5–6.1 months) and the non-HRD group (4.6 months, 95% CI: 4.4–5.1 months) in the GnP cohort (*P* = 0.44, Fig. 1b).

**Table 4:** Best of overall response

	Total	HRD	Non-HRD	
Total	*N* = 1394	*N* = 107	*N* = 1287	*P* value
CR/PR/SD/PD/NE	2/459/534/313/86	0/42/35/20/10	2/417/499/293/76	0.25
ORR	461 (33)	42 (39)	419 (33)	0.17
DCR	995 (71)	77 (72)	918 (71)	1.00

	Total	HRD	Non-HRD	
FFX	*N* = 437	*N* = 34	*N* = 403	*P* value
CR/PR/SD/PD/NE	1/145/143/126/22	0/19/7/6/2	1/126/136/120/20	0.05
ORR	146 (33)	19 (56)	127 (32)	0.01
DCR	289 (66)	26 (77)	263 (65)	0.26

	Total	HRD	Non-HRD	
GnP	*N* = 957	*N* = 73	*N* = 884	*P* value
CR/PR/SD/PD/NE	1/314/391/187/64	0/23/28/14/8	1/291/363/173/56	0.54
ORR	315 (33)	23 (32)	292 (33)	0.90
DCR	706 (74)	51 (70)	655 (74)	0.41

*CR* complete responses, *PR* partial responses, *SD* stable responses, *PD* progressive disease, *NE* not evaluated, *ORR* overall response rate, *DCR* disease control rate, *GnP* gemcitabine and nab-paclitaxel, *FFX* FOLFIRINOX, *HRD* homologous recombination deficiency

**Fig. 2:**
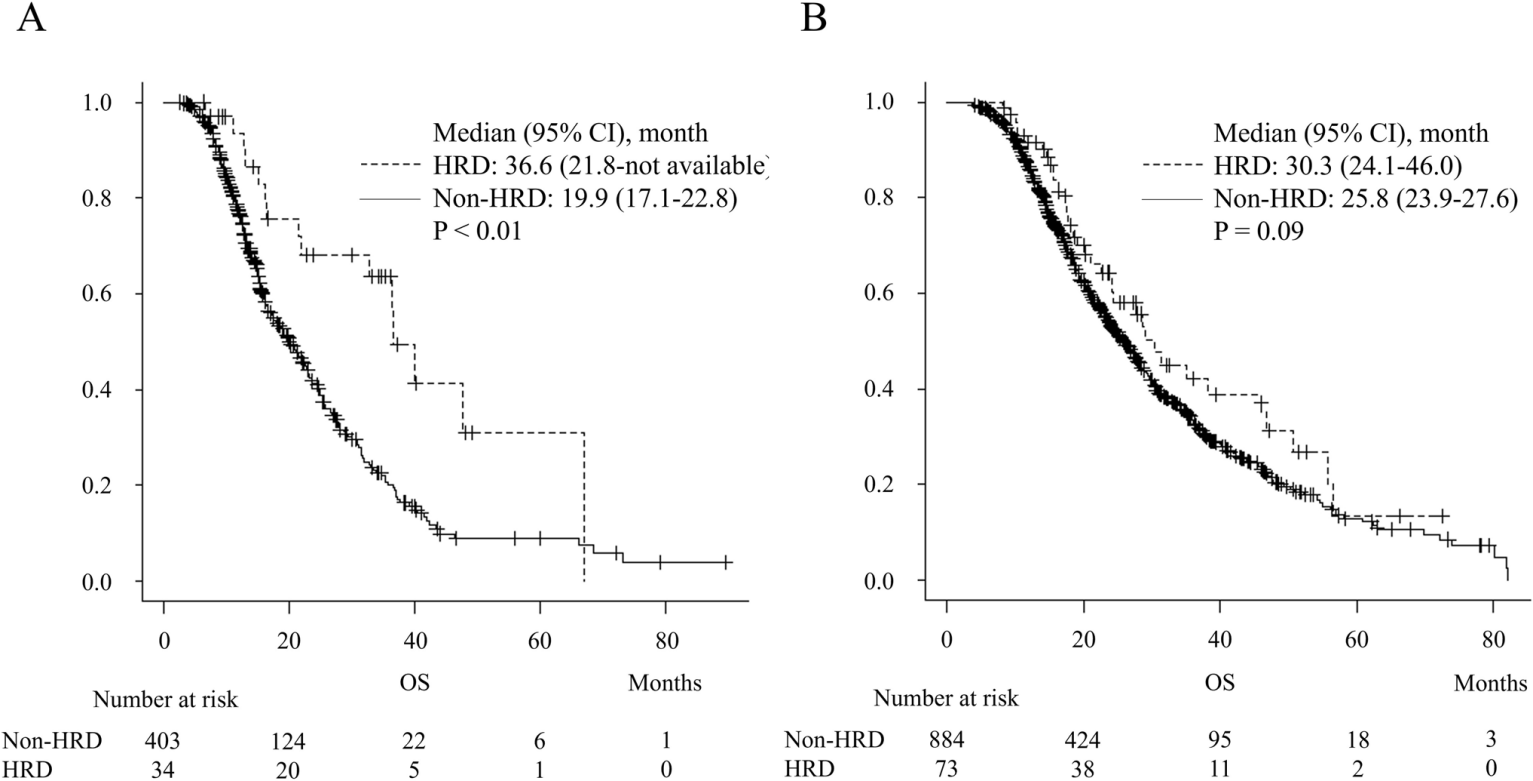
Comparison of OS in FFX and GnP Cohort (HRD vs non-HRD). **A** In the FFX cohort, the median OS was significantly longer in the HRD group, with a median of 36.6 months (95% CI: 21.8-not available), compared with 19.9 months (95% CI: 17.1–22.8) in the non-HRD group (*P* < 0.01). **B** In the GnP cohort, the median OS was comparable between the HRD group, with a median of 30.3 months (95% CI: 24.1–46), and the non-HRD group, with a median of 25.8 months (95% CI: 23.9–27.6). Therefore, this showed no significant difference between the two groups. *OS* overall survival, *FFX* FOLFIRINOX, *GnP* gemcitabine and nab-paclitaxel, *HRD* homologous recombination deficiency, *CI* confidence interval

In the FFX cohort, the median OS was significantly longer in the HRD group, with a median of 36.6 months (95% CI: 21.8—not available), compared with 19.9 months (95% CI: 17.1–22.8) in the non-HRD group (*P* < 0.01, Fig. 2a). In the GnP cohort, the median OS was comparable between the HRD group, with a median of 30.3 months (95% CI: 24.1–46.0), and the non-HRD group, with a median of 25.8 months (95% CI: 23.9–27.6), showing no significant difference between the two groups (*P* = 0.09, Fig. 2b).

When comparing the median OS of FFX and GnP in the HRD group, no significant difference was observed between the two groups (36.6 months vs 30.3 months, *P* = 0.50).

### An analysis focused on genetic abnormalities with a high GCR

**Fig. 3:**
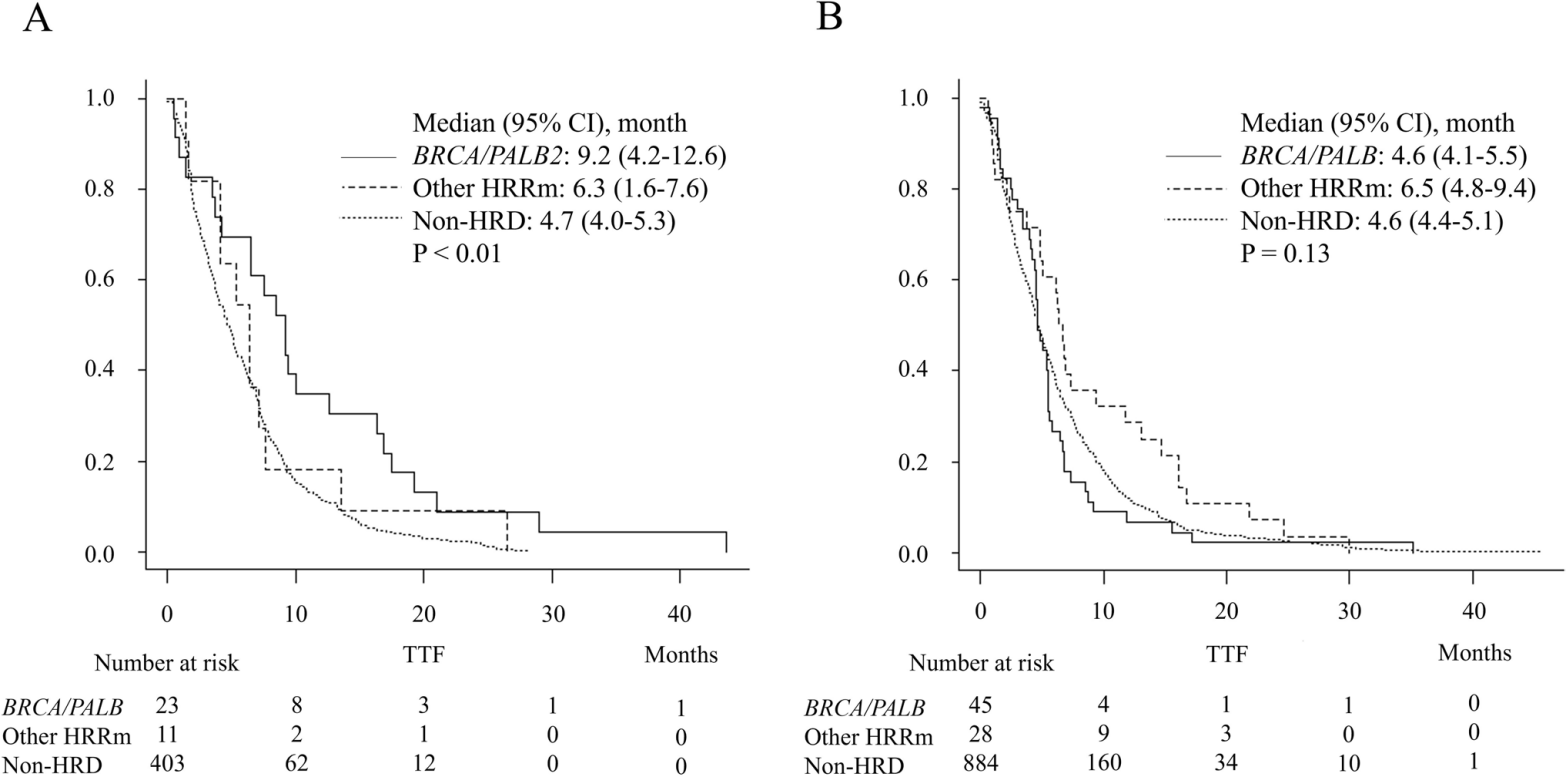
Comparison of TTF in FFX and GnP Cohort (*BRCA/PALB* group vs Other HRRm group vs non-HRD group). **A** In the FFX cohort, the median TTF was significantly longer in the *BRCA/PALB* group, with a median of 9.2 months (95% CI: 4.2–12.6), compared with 6.3 months (95% CI: 1.6–7.6) in the Other HRRm group, and 4.7 months (95% CI: 4.0–5.3) in the non-HRD group (*P* < 0.01). **B** In the GnP cohort, the median TTF was comparable between the three groups (*P* = 0.13). *TTF* time-to-treatment failure, *FFX* FOLFIRINOX, *GnP* gemcitabine and nab-paclitaxel, *HRRm* homologous recombination repair-related gene mutations, *HRD* homologous recombination deficiency, *CI* confidence interval

In the FFX cohort, the median TTF was significantly longer in the *BRCA*/*PALB* group, with a median of 9.2 months (95% CI: 4.2–12.6), compared with 6.3 months (95% CI: 1.6–7.6) in the Other HRRm group, and 4.7 months (95% CI: 4–5.3) in the non-HRD group (*P* < 0.01, Fig. 3a). In the GnP cohort, the median TTF was comparable between the three groups (*P* = 0.13, Fig. 3b).

## Discussion

In the analysis of the C-CAT database, we found 7.6% of PC undergoing CGP tests exhibited HRD. HRD was associated with a higher ORR and longer TTF specifically in the FFX cohort, but this association was not observed in the GnP cohort. This suggests HRD can be a predictive factor of effectiveness of platinum-based regimens in PC. Regarding OS, in the FFX cohort, the HRD group showed significantly better outcomes. In the GnP cohort, although there was no statistically significant difference, the HRD group also showed better outcomes. This may be attributed to the fact that platinum-based regimens were used in 53% of cases as second-line and later treatments; when limited to the HRD group, platinum regimens were used in 59% of cases as second-line and later treatments. Both GnP and FFX are established as standard of treatment for patients with unresectable and recurrent PC. However, due to the survival superiority of GnP over FFX demonstrated in the GENERATE trial [16], GnP is expected to become the primary choice for first-line treatment in Japan. Nevertheless, our study results propose that FFX might be favored over GnP, particularly in patients exhibiting HRD.

It is already known that platinum-based regimens are effective against solid tumors with HRD [23, 24] and the PARP inhibitor olaparib is approved for insurance coverage in PC with germline *BRCA1/2* mutations [17]. In addition to *BRCA1/2*, which are well-known genes strongly associated with HRD, we analyzed ten other genes as HRR-related genes. When dividing the HRD group into the *BRCA/PALB* group and the Other HRRm group, notable variances in TTF were detected among different subgroups in the FFX cohort. In contrast, in the GnP cohort, no distinct difference in TTF were observed among different subgroups. Genes with low GCR often exhibit tumor-derived abnormalities in HRR-related genes, requiring two hits to become clinically significant HRD. However, in this study, since we did not assess variant allele frequencies, evaluating whether it was one hit or two hits was insufficient. Meanwhile, when abnormalities in HRR-related genes occur in the germline, there is a high possibility that the cancer has developed after a second hit [25, 26], making it more likely to be clinically significant HRD and substantially influencing the outcomes of platinum-based regimens.

In PC, it is crucial to not only identify *BRCA1/2* mutations but also *PALB2* mutations. This is because mutations in *BRCA1/2* and *PALB2* are predominantly germline mutations, and PC with these mutations is highly likely to exhibit clinically significant HRD. While BRACAnalysis^®^ [27] is available in unresectable PC patients, *PALB2* cannot be examined using the BRACAnalysis^®^. When CGP tests are indicated only for patients who have completed standard treatment as is the case in Japan, it remains challenging to identify patients with HRD before initiating first-line treatment. Prediction based on family history is the most straightforward method [28]. Moreover, the presence of HRR-related gene mutations significantly correlated with a family history of HRD-related cancers in individuals under 50 years old and with a family history of two or more HRD-related cancers within third-degree relatives in the analysis of the C-CAT database. The patients with such strong family histories may be candidates for platinum-based regimens, suggesting that this could be a strategic approach in the future.

This study has several limitations. First, the C-CAT database contains some missing or inaccurate data. However, we included only cases with available TTF, OS, and ORR for analysis. As a result, the final proportion of missing data was minimal, ranging from 0.1 to 0.3%. Given the large sample size of this study, the impact of the missing data on the analyses is likely negligible. In addition, as mentioned above, the mere presence of HRR-related genetic abnormalities is not enough to accurately assess clinically significant HRD. Moreover, the results of this study seem to be significantly influenced by mutations in *BRCA1/2* and *PALB2*. Since there are only 11 cases with mutations in HRR-related genes other than *BRCA1/2* and *PALB2*, it is necessary to accumulate more cases to determine the extent of their influence on the efficacy of platinum-based regimens. Lastly, reports have already shown that NALIRIFOX has significantly better OS compared with GnP [29]. Therefore, once NALIRIFOX becomes available in clinical practice, it can be a first-line chemotherapy for unresectable pancreatic cancer, irrespective of the HRD status.

In conclusion, HRR-related genetic abnormalities might be predictive of TTF in platinum-based chemotherapy for PC. Further investigation is needed to determine how HRD is picked up and which platinum regimens are used.

## Supplementary Information

Below is the link to the electronic supplementary material.

Supplementary file1 (DOCX 26 KB)

Supplementary file2 (DOCX 25 KB)

## Abbreviations

C-CAT: Center for Cancer Genomics and Advanced Therapeutics
CGP: Comprehensive genomic profiling
CHIP: Clonal hematopoiesis of indeterminate potential
CI: Confidence intervals
CR: Complete response
DCR: Disease control rate
FFX: FOLFIRINOX
F1CDx: FoundationOne^®^ CDx
GCR: Germline conversion rate
GnP: Gemcitabine and nab-paclitaxel
HRD: Homologous recombination deficiency
HRR: Homologous recombination repair
MSI: Microsatellite instability
NOP: Oncoguide^™^ NCC oncopanel
ORR: Overall response rate
OS: Overall survival
PARP: Poly ADP-ribose polymerase
PC: Pancreatic cancer
PD: Progressive disease
PR: Partial response
SD: Stable disease
TMB: Tumor mutational burden
TTF: Time to treatment failure

## References

[1] Ebi H, Bando H. Precision oncology and the universal health coverage system in Japan. JCO Precis Oncol. 2019. 10.1200/PO.19.00291. 32923862 10.1200/PO.19.00291 PMC7446489

[2] Sunami K, Naito Y, Komine K, et al. Chronological improvement in precision oncology implementation in Japan. Cancer Sci. 2022;113:3995–4000. 35976133 10.1111/cas.15517 PMC9633287

[3] Kohno T, Kato M, Kohsaka S, et al. C-CAT: The National Datacenter for Cancer Genomic Medicine in Japan. Cancer Discov. 2022;12:2509–15. 36321305 10.1158/2159-8290.CD-22-0417 PMC9762342

[4] Noji R, Tohyama K, Kugimoto T, et al. Comprehensive genomic profiling reveals clinical associations in response to immune therapy in head and neck cancer. Cancers (Basel). 2022;14:3476. 35884537 10.3390/cancers14143476 PMC9315472

[5] Xi Q, Kage H, Ogawa M, et al. Genomic landscape of endometrial, ovarian, and cervical cancers in Japan from the database in the center for cancer genomics and advanced Therapeutics. Cancers (Basel). 2023;16:136. 38201563 10.3390/cancers16010136 PMC10778092

[6] Kurokawa K, Shukuya T, Greenstein RA, et al. Genomic characterization of thymic epithelial tumors in a real-world dataset. ESMO Open. 2023;8: 101627. 37703595 10.1016/j.esmoop.2023.101627 PMC10594028

[7] Saito Y, Kage H, Kobayashi K, et al. TERT promoter mutation positive oral cavity carcinomas, a clinically and genetically distinct subgroup of head and neck squamous cell carcinomas. Head Neck. 2023;45:3107–18. 37815002 10.1002/hed.27540

[8] Sakakida T, Ishikawa T, Doi T, et al. Genomic landscape and clinical features of rare subtypes of pancreatic cancer: analysis with the national database of Japan. J Gastroenterol. 2023;58:575–85. 37029223 10.1007/s00535-023-01986-9 PMC10199859

[9] Kobayashi K, Saito Y, Kage H, et al. CDK12 alterations and ARID1A mutations are predictors of poor prognosis and therapeutic targets in high-grade salivary gland carcinoma: analysis of the National Genomic Profiling Database. Jpn J Clin Oncol. 2023;53:798–807. 37357968 10.1093/jjco/hyad066

[10] Otani R, Ikegami M, Yamada R, et al. PTPN11 variant may be a prognostic indicator of IDH-wildtype glioblastoma in a comprehensive genomic profiling cohort. J Neurooncol. 2023;164:221–9. 37552362 10.1007/s11060-023-04411-6

[11] Naito T, Noji R, Kugimoto T, et al. The efficacy of immunotherapy and clinical utility of comprehensive genomic profiling in adenoid cystic carcinoma of head and neck. Medicina (Kaunas). 2023;59:2111. 38138214 10.3390/medicina59122111 PMC10745089

[12] Sakakida T, Ishikawa T, Doi T, et al. Genomic profile and clinical features of MSI-H and TMB-high pancreatic cancers: real-world data from C-CAT database. J Gastroenterol. 2024;59:145–56. 38006445 10.1007/s00535-023-02058-8

[13] Kobayashi H, Zhang L, Okajima K, et al. BRAF mutations and concurrent alterations in patients with soft tissue sarcoma. Genes Chromosomes Cancer. 2023;62:648–54. 37293958 10.1002/gcc.23182

[14] Von Hoff DD, Ervin T, Arena FP, et al. Increased survival in pancreatic cancer with nab-paclitaxel plus gemcitabine. N Engl J Med. 2013;369:1691–703. 24131140 10.1056/NEJMoa1304369 PMC4631139

[15] Conroy T, Desseigne F, Ychou M, et al. FOLFIRINOX versus gemcitabine for metastatic pancreatic cancer. N Engl J Med. 2011;364:1817–25. 21561347 10.1056/NEJMoa1011923

[16] Ohba AOM, Ogawa G. 1616O Nab-paclitaxel plus gemcitabine versus modified FOLFIRINOX or S-IROX in metastatic or recurrent pancreatic cancer (JCOG1611, GENERATE): a multicentred, randomized, open-label, three-arm, phase II/III trial. Ann Oncol. 2023;34:S894.

[17] Golan T, Hammel P, Reni M, et al. Maintenance olaparib for germline BRCA-mutated metastatic pancreatic cancer. N Engl J Med. 2019;381:317–27. 31157963 10.1056/NEJMoa1903387 PMC6810605

[18] Seeber A, Zimmer K, Kocher F, et al. Molecular characteristics of BRCA1/2 and PALB2 mutations in pancreatic ductal adenocarcinoma. ESMO open. 2020;5: e000942. 33229504 10.1136/esmoopen-2020-000942 PMC7684832

[19] O’Reilly EM, Lee JW, Zalupski M, et al. Randomized, multicenter, phase II trial of gemcitabine and cisplatin with or without veliparib in patients with pancreas adenocarcinoma and a germline BRCA/PALB2 mutation. J Clin Oncol. 2020;38:1378–88. 31976786 10.1200/JCO.19.02931 PMC7193749

[20] Wattenberg MM, Asch D, Yu S, et al. Platinum response characteristics of patients with pancreatic ductal adenocarcinoma and a germline BRCA1, BRCA2 or PALB2 mutation. Br J Cancer. 2020;122:333–9. 31787751 10.1038/s41416-019-0582-7 PMC7000723

[21] Galland L, Roussot N, Desmoulins I, et al. Clinical utility of genomic tests evaluating homologous recombination repair deficiency (HRD) for treatment decisions in early and metastatic breast cancer. Cancers (Basel). 2023;15:1299. 36831640 10.3390/cancers15041299 PMC9954086

[22] Kuzbari Z, Bandlamudi C, Loveday C, et al. Germline-focused analysis of tumour-detected variants in 49,264 cancer patients: ESMO Precision Medicine Working Group recommendations. Ann Oncol. 2023;34:215–27. 36529447 10.1016/j.annonc.2022.12.003

[23] Pokataev I, Fedyanin M, Polyanskaya E, et al. Efficacy of platinum-based chemotherapy and prognosis of patients with pancreatic cancer with homologous recombination deficiency: comparative analysis of published clinical studies. ESMO open. 2020;5: e000578. 33551067 10.1136/esmoopen-2019-000578 PMC7003386

[24] Chen KT, Madison R, Moore J, et al. A novel HRD signature is predictive of FOLFIRINOX benefit in metastatic pancreatic cancer. Oncologist. 2023;28:691–8. 37354528 10.1093/oncolo/oyad178 PMC10400136

[25] Chenevix-Trench G, Healey S, Lakhani S, et al. Genetic and histopathologic evaluation of BRCA1 and BRCA2 DNA sequence variants of unknown clinical significance. Cancer Res. 2006;66:2019–27. 16489001 10.1158/0008-5472.CAN-05-3546

[26] Meric-Bernstam F. Heterogenic loss of BRCA in breast cancer: the “two-hit” hypothesis takes a hit. Ann Surg Oncol. 2007;14:2428–9. 17406948 10.1245/s10434-007-9379-7

[27] Gunderson CC, Moore KN. BRACAnalysis CDx as a companion diagnostic tool for Lynparza. Expert Rev Mol Diagn. 2015;15:1111–6. 26292709 10.1586/14737159.2015.1078238

[28] Okano N, Morizane C, Nomura S, et al. Phase II clinical trial of gemcitabine plus oxaliplatin in patients with metastatic pancreatic adenocarcinoma with a family history of pancreatic/breast/ovarian/prostate cancer or personal history of breast/ovarian/prostate cancer (FABRIC study). Int J Clin Oncol. 2020;25:1835–43. 32535711 10.1007/s10147-020-01721-x

[29] Wainberg ZA, Melisi D, Macarulla T, et al. NALIRIFOX versus nab-paclitaxel and gemcitabine in treatment-naive patients with metastatic pancreatic ductal adenocarcinoma (NAPOLI 3): a randomised, open-label, phase 3 trial. Lancet. 2023;402:1272–81. 37708904 10.1016/S0140-6736(23)01366-1 PMC11664154

