# Supplementary material for: Association between homologous recombination deficiency and time to treatment failure to platinum-based chemotherapy for pancreatic cancer by using the C-CAT database
Source: J Gastroenterol. 2024 Nov 21;60(2):247–56. doi: 10.1007/s00535-024-02173-0 (PMC11794350; doi:10.1007/s00535-024-02173-0)
Supplement: Supplementary file 1 — Supplementary file1 (DOCX 26 KB) [file 535_2024_2173_MOESM1_ESM.docx]

**Supplementary table 1. Details of second-line and later treatments**

|  | Total | HRD | Non-HRD |  |
| --- | --- | --- | --- | --- |
|  | N = 1394 | N = 107 | N = 1287 | P value |
| Introduction of second-line treatment, n (%) |  |  |  |  |
| Total cohort | 1255 (90) | 96 (90) | 1159 (90) | 0.87 |
| FFX | 390 (89) | 28 (82) | 362 (90) | 0.24 |
| GnP | 865 (90) | 68 (93) | 797 (90) | 0.54 |
| Introduction of platinum regimen in second-line and later treatments, n (%) |  |  |  |  |
| Total cohort | 538 (39) | 48 (45) | 490 (38) | 0.24 |
| FFX | 34 (8) | 5 (15) | 29 (7) | 0.17 |
| GnP | 504 (53) | 43 (59) | 461 (52) | 0.33 |

FFX; FOLFIRINOX, GnP; gemcitabine and nab-paclitaxel, HRD; homologous recombination deficiency.
