# Supplementary material for: Association between homologous recombination deficiency and time to treatment failure to platinum-based chemotherapy for pancreatic cancer by using the C-CAT database
Source: J Gastroenterol. 2024 Nov 21;60(2):247–56. doi: 10.1007/s00535-024-02173-0 (PMC11794350; doi:10.1007/s00535-024-02173-0)
Supplement: Supplementary file 2 — Supplementary file2 (DOCX 25 KB) [file 535_2024_2173_MOESM2_ESM.docx]

**Supplementary Table 2. Family history of HRD-related cancer**

|  | Total | HRD | Non-HRD |  |
| --- | --- | --- | --- | --- |
|  | N = 1394 | N = 107 | N = 1287 | P value |
| Family history of HRD-related cancer | 437 (31.4) | 44 (41.1) | 393 (30.5) | 0.03 |
| Family history of pancreatic or ovarian cancer | 223 (16) | 24 (22.4) | 199 (15.5) | 0.07 |
| Family history of HRD-related cancer in one or more first-degree relatives | 289 (20.7) | 26 (24.3) | 263 (20.4) | 0.38 |
| Family history of HRD-related cancer in two or more third-degree relatives | 87 (6.2) | 16 (15.0) | 71 (5.5) | < 0.01 |
| Family history of HRD-related cancer under age of 50 years old | 74 (5.3) | 13 (12.1) | 61 (4.7) | < 0.01 |

HRD; homologous recombination deficiency.
